# Ultrathin Organic Solar Cells with a Power Conversion Efficiency of Over ≈13.0%, Based on the Spatial Corrugation of the Metal Electrode–Cathode Fabry–Perot Cavity

**DOI:** 10.1002/advs.201700900

**Published:** 2018-01-31

**Authors:** Sungjun In, Namkyoo Park

**Affiliations:** ^1^ Department of Electrical and Computer Engineering Seoul National University Seoul 08826 South Korea

**Keywords:** corrugated cavities, device physics, electrical transport, near‐perfect spectral overlap, organic solar cells

## Abstract

The application of nanophotonic structures for organic solar cells (OSCs) is quite popular and successful, and has led to increased optical absorption, better spectral overlap with solar irradiances, and improved charge collection. Significant improvements in the power conversion efficiency (PCE) have also been reported, exceeding 11%. Nonetheless, with the given material properties of OSCs with low optical absorption, narrow spectrum, short transport length of carriers, and nonuniform photocarrier generations resulting from the nanophotonic structure, the PCE of single‐junction OSCs has been stagnant over the past few years, at a barrier of 12%. Here, an ultrathin inverted OSC structure with the highest efficiency of ≈13.0%, while being made from widely used organic materials, is demonstrated. By introducing a smooth spatial corrugation to the vertical plasmonic cavity enclosing the active layer, in‐plane propagation modes and hybridized Fabry–Perot cavity modes inside the corrugated cavity are derived to achieve an ultralow *Q*, uniform coverage of optical absorption, in addition to uniform photocarrier generation and transport. As the first demonstration of ultra‐broadband absorption with the introduction of spatial corrugation to the ultrathin metal film electrode–cathode Fabry–Perot cavity, future applications of the same concept in other light‐harvesting devices utilizing different materials and structures are expected.

## 1 Introduction

Significant improvements have been made in the performance of organic solar cells (OSCs) with the introduction of nanophotonic effects,^1^, ^2^, ^3^, ^4^, ^5^, ^6^, ^7^, ^8^ material‐interface synthesis,^9^, ^10^ and the development of high‐performing organic materials.^11^, ^12^ The state‐of‐the‐art single‐junction OSCs with plasmonic cavity structures,^1^, ^2^, ^3^ textured light trapping structures,^4^, ^5^ multiplasmonic effects,^6^, ^7^, ^8^ morphological implementation,^9^, ^10^ bandgap tuning,^11^, ^12^ and solution fabrication method^13^ now provide over 8–11% of power conversion efficiency (PCE) based on the above approaches or a combination of these approaches. However, over the past few years, a serious difficulty has also been observed in the increase of the PCE of single‐junction OSCs to over 12%. The narrow absorption band of organic materials, incomplete spectral engineering matching to the absorption band of the active layer, recombination losses from low charge carrier mobilities, and nonuniform photocarrier generation/transport caused by subwavelength nanostructures together comprise the current bottleneck and therefore an opportunity in PCE improvement.

Even though some of the above‐mentioned issues have been mediated with the introduction of new materials such as poly[(2,6‐(4,8‐bis(5‐(2‐ethylhexyl)thiophen‐2‐yl)benzo[1,2‐b:4,5‐b′]dithiophene)‐*co*‐(1,3‐di(5‐thiophene‐2‐yl)‐5,7‐bis(2‐ethylhexyl)benzo[1,2‐c:4,5‐c′]dithiophene‐4,8‐dione)]:3,9‐bis(2‐methylene‐(3‐(1,1‐dicyanomethylene)‐indanone))‐5,5,11,11‐tetrakis(4‐hexylphenyl)‐dithieno[2,3‐d:2′,3′‐d′]‐s‐indaceno[1,2‐b:5,6‐b′]‐dithiophene) modified acceptor (PBDB:IT‐M), providing wider absorption band and larger open‐circuit voltage (achieving a PCE of ≈12.1%), the comprehensive engineering of the OSC considering both the ideal spectral engineering and electrical carrier collection is still rare. At the current stage, no report has been presented for single‐junction OSCs providing broadband and uniform optical absorption yet within an ultrathin active layer (≈100 nm), while employing widely used organic materials. In addition, current reports repeatedly show a significant reduction in the fill factors (FF) of nanophotonic structure‐based OSCs (e.g., plasmonic nanograting,^14^ bumped‐structure,^15^ and nanoparticle^16^), despite their improved PCE. Even if many prior arts also relate the dependence of FF to the active layer thickness and electrical carrier transport/collection,^13^, ^17^, ^18^ a systematic understanding is still incomplete, considering the interplay between the participating dynamics.

In this paper, we show the feasibility of an organic solar cell of near ≈13% PCE, in inverted configurations of widely used organic materials of poly((4,8‐bis[(2‐ethylhexyl)oxy]benzo[1,2‐b:4,5‐b′]dithiophene‐2,6‐diyl)(3‐fluoro‐2‐[(2‐ethylhexyl)‐carbonyl]‐thieno[3,4‐b]thiophenediyl)):[6,6]‐phenyl‐C_71_‐butyric acid methyl ester (PBDTT‐F‐TT:PC_71_BM). First, working on a simple structure of ultrathin metal film (UTMF)‐electrode OSC by employing the optical–electrical multiphysics analysis, we reveal that it is the spectral engineering, perfecting the absorption band of the active layer, not the (electrical) mobility, which defines the present device performances bottleneck of OSC. Then, by introducing a smooth spatial corrugation to the active layer cavity of the UTMF‐electrode OSC, without any penalty to electrical transports, we derive a strong multipeak in‐plane plasmonic propagation modes in addition to the broadening of the Fabry–Perot cavity modes in the UTMF‐electrode OSC: in order to achieve highly uniform (>85%) and ultralow *Q* (330–775 nm) absorption, which provide near‐perfect spectral overlap to the ultrathin (120 nm) PBDTT‐F‐TT:PC_71_BM active layer. Equally importantly, based on the drift current distributions from the optical–electrical multiphysics modeling, we also show that the proposed corrugated cavity structure provides excellent electrical scale length, with negligible scattering. Detailed analysis is carried out on the influence of the corrugated cavity to the optical field hybridization, exciton generation rate, charge carrier collection efficiency, and electrical conversion efficiency, in order to provide deeper insight into the origin of full‐visible optical absorption, and thus to enlighten a systematic pathway in the tuning of electrical performance of OSCs.

## 2 Results and Discussion

### 2.1 Performance Analysis of Reference OSC: Case of Flat UTMF OSC

**Figure 1:**
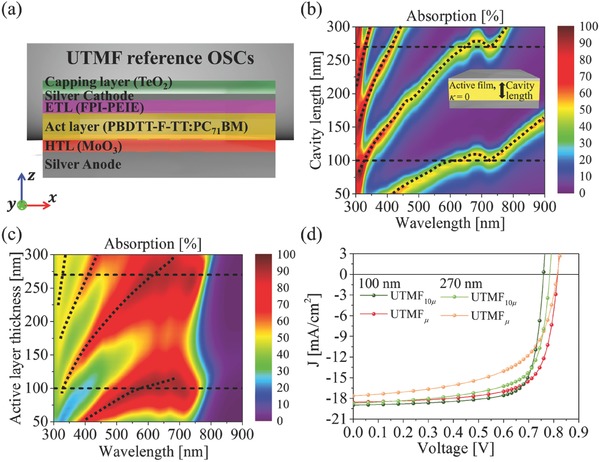
Photovoltaic performance of control device: flat UTMF OSC. a) Schematic of flat UTMF OSC. b) Optical absorption map of UTMF cavity filled with dispersive PBDTT‐F‐TT:PC_71_BM film (while neglecting material absorption, κ = 0) as a function of wavelength (*x*‐axis) and cavity length (*y*‐axis). c) Optical absorption map of flat UTMF OSC filled with PBDTT‐F‐TT:PC_71_BM active layer. d) *J–V* (current density–voltage) characteristics of device at different active layer thicknesses (100 and 270 nm) and carrier mobilities. For all data, AM1.5G‐weighted, normally incident plane‐wave illumination was assumed.

First, as a control reference (**Figure** **1**a), we consider flat UTMF‐electrode OSCs of near‐perfect optical absorption providing a PCE of 8–11%.^1^, ^3^, ^6^ In order to investigate the key factors in determining the PCE of the flat UTMF‐electrode OSCs, we first analyze the optical absorption map of the reference UTMF‐cavity as a function of wavelength and cavity length. Figure 1b (cavity filled with PBDTT‐F‐TT:PC_71_BM, while neglecting material absorption, κ = 0) and Figure 1c (cavity filled with OSC compositions and capping layers) each shows the clear dependence of Fabry–Perot resonances for the increase of cavity length. In addition, comparing Figure 1b and Figure 1c, we also note that, in the case of the PBDTT‐F‐TT:PC_71_BM‐filled cavity, strong absorption at the large material absorption wavelength (550–700 nm) is obtained at the active layer thickness of 100 nm, concurring with previous experimental reports,^1^ demonstrating the need for material‐absorption‐weighted spectral engineering in the determination of the device structure and its resonances. It is worth mentioning as well, the other optimal cavity length at 270 nm shows much lower incident photon to current conversion efficiency (IPCE) values than the 100 nm cavity (shown in Figure S1, Supporting Information), due to the increased charge transport length and thus reduced optical–electrical conversion.

Clarified the importance of optical absorption, in terms of material‐absorption‐weighted device resonance engineering, we now investigate the electrical effect of carrier mobility on the power conversion efficiency of OSC. For this purpose, we perform coupled optical–electrical analysis,^19^, ^20^, ^21^, ^22^, ^23^ assuming different values of mobility: at *µ*_e,h_, and also at a much larger value of 10 *µ*_e,h_ (*µ*_h_ = 3.58 × 10^−4^ cm^2^ V^−1^ S^−1^ and *µ*_e_ = 3.42 × 10^−4^ cm^2^ V^−1^ S^−1^ of PBDTT‐F‐TT:PC_71_BM^24^, ^25^). Figure 1d presents the *J–V* characteristic of the UTMF‐electrode reference OSCs, at different combinations of active layer thickness (100 and 270 nm) and mobility values (*µ*_e,h_, 10 *µ*_e,h_). First, for an inverted OSC of 100 nm active layer with *µ*_e,h_ (green lines), a PCE of 10.9%, an open‐circuit voltage of 0.815 V, a short‐circuit current of 18.57 mA cm^−2^, and a fill factor (FF; *J*_max_ *V*_max_/*J*_sc_ *V*_oc_) of 71.7% were obtained, in good agreement with previous experimental reports.^1^

By increasing the carrier mobility from *µ*_e,h_ to 10 *µ*_e,h_, improved FF values were obtained at each fixed active layer thickness (71.7–76.2% for 100 nm and 59.3–69.2% for 270 nm), as expected. Nonetheless, since the 100 nm active layer device with *µ*_e,h_ provides better FF (Figure 1d) and PCE (Figure S2, Supporting Information) than those of the 270 nm device of 10 *µ*_e,h_, we conclude that the carrier recombination provides a negligible contribution to the PCE increase, and the effort for higher PCE should be focused toward an increased optical absorption. Most importantly, noting that the narrowband Fabry–Perot cavity resonance (Figure 1b) covers only the partial spectrum of PBDTT‐F‐TT:PC_71_BM absorption, we find it is critical to hybridize the narrowband cavity mode with another broadband, or multiples of modes residing in the structure.

### 2.2 Performance Analysis of Proposed OSC: Case of Corrugated Cavity UTMF OSC

**Figure 2:**
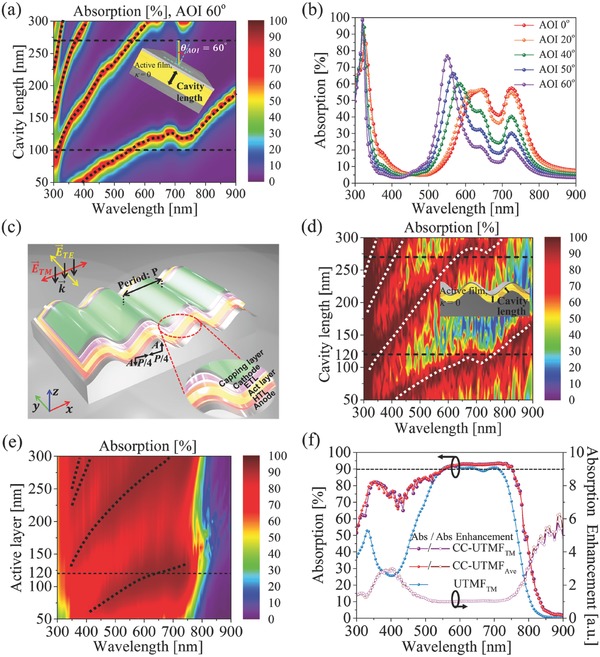
Optical characteristics of corrugated cavity UTMF (CC‐UTMF) OSC. a) Optical absorption map of UTMF‐cavity structure filled with PBDTT‐F‐TT:PC_71_BM (while neglecting material absorption), as a function of wavelength (*x*‐axis) and cavity length (*y*‐axis). Angle of incidence = 60°. b) Optical absorption spectra at different AOI (0°–60°). c) Schematic of the CC‐UTMF OSCs. *P* = 8400 nm, *P*/4 = 2100 nm, *A* = 1200 nm. d) Optical absorption map for corrugated cavity (filled with PBDTT‐F‐TT:PC_71_BM, while neglecting material absorption, κ = 0) as a function of wavelength (*x*‐axis) and cavity length (*y*‐axis). e) Optical absorption map of corrugated cavity UTMF OSC (filled with PBDTT‐F‐TT:PC_71_BM). f) Optical absorption spectra in the active layer (solid symbols) and optical absorption enhancement (open symbols) of CC‐UTMF, relative to the control UTMF absorption (blue), (dashed horizontal line marking 90%).

To derive a uniform coverage of optical absorption overcoming the narrowband cavity mode, we first investigate the spectral response of the reference UTMF‐cavity under different angle of incidence (AOI). **Figure** **2**a shows the optical absorption map of UTMF‐cavity at an AOI of 60°, while Figure 2b illustrates the absorption spectra of UTMF‐cavity as a function of AOI at a fixed cavity of 100 nm. With the increase in AOI (0°–60°), the blueshift of the Fabry–Perot resonances is evident, due to the reflection phase shift caused by the increase of effective path length^26^, ^27^ in UTMF. Based on the above observations we introduce spatial corrugation to the Fabry–Perot cavity in UTMF OSC (Figure 2c), in order to achieve broader absorption from the contributions of different AOI absorption bands and corrugation‐excited propagation modes.^28^, ^29^, ^30^, ^31^, ^32^, ^33^ The composed OSC assumes identical material compositions as those of the reference OSCs, except for the round‐sine shape corrugation (for fabrication methods, see Experimental Section). It is noted that the modulation period (*P*) is set to be much larger than that of conventional sub‐wavelength gratings^8^, ^34^, ^35^, ^36^ with a small amplitude/period ratio (*A*/*P*), in order to retain a small modulation‐induced wave‐vector perturbation, *k_z_*. This ensures the local Fabry–Perot resonance condition remains intact, while promoting the hybridization with the multipeak in‐plane propagation modes residing inside the active layer.

Figure 2d (corrugated cavity filled with PBDTT‐F‐TT:PC_71_BM, κ = 0) and Figure 2e (corrugated cavity with OSC compositions and capping layers) both show the plot of optical absorption as a function of cavity length and incident wavelength. For both figures, in comparison with the flat cavities shown in Figure 1b,c, the broadening of the cavity mode from the contributions of different AOI absorption bands is evident, in addition to the emergence of the in‐plane propagation modes in the short wavelength regime.^30^, ^31^, ^32^, ^33^ It is worth mentioning that the calculated optical absorption was relatively insensitive to the variation of height and period of the corrugation, over the range of 1000 nm <*P*/4 < 2500 nm and 1000 nm < *A* < 2500 nm (see the Supporting Information).

Figure 2f also shows the optical absorption and absorption enhancement ratio in the active layer of CC‐UTMF (corrugated cavity UTMF) OSC, calculated for both the optically‐ and electrically optimized OSC structure (active layer thickness = 120 nm), under the normal incident light (AM1.5G solar spectrum). The absorption of the proposed CC‐UTMF OSCs is considerably increased when compared to the UTMF reference flat‐cavity OSCs, achieving ultralow *Q* (330–775 nm) and highly uniform (>85%) strong optical absorption, stretching from the near UV to the near IR range. Compared to the UTMF‐electrode reference cell, the AM1.5 weight‐averaged figure of merit (see the Supporting Information) in the optical absorption was calculated to have increased by ≈21.5%. It is emphasized that this large enhancement of optical absorption signifying the contribution from the corrugated cavity, directly translates to the huge increase of short‐circuit current (discussed later with Figure 4).

**Figure 3:**
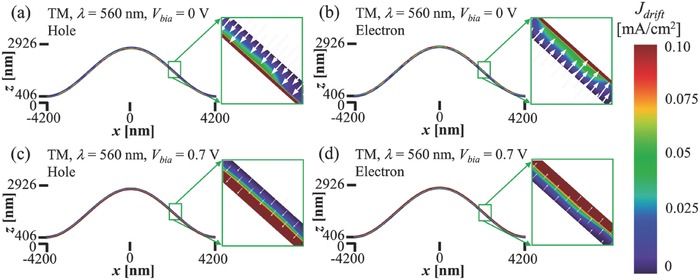
Drift current density and current flow of the CC‐UTMF OSC under short‐circuit current (*V*_bias_ = 0 V) and near open‐circuit voltage (*V*_bias_ = 0.7 V) condition. a,c) Hole and b,d) electron drift current density (color) and current flow (white arrows) of CC‐UTMF, under TM polarization illumination at λ = 560 nm.

After verifying the highly uniform and ultralow *Q* optical absorption with the introduction of the corrugated cavity, which provides perfect spectral overlap for the ultrathin active layer, we now investigate the effect of the corrugated cavity on the charge transport dynamics. In order to analyze the charge transport and collection, we focus on the distribution of the drift current densities and current flow in the active layer. **Figure** **3** shows the hole (or electron) drift currents under TM illumination (see Figure S7 for TE in the Supporting Information), at short‐circuit current (*V*_bias_ = 0 V, Figure 3a,b) and near the open‐circuit condition (*V*_bias_ = 0.7 V, Figure 3c,d). When compared to the current densities of the reference device (shown in Figure S8 in the Supporting Information), those of similar distribution and magnitude are observed for both bias conditions (except the minor signature of the modal profile in the distribution of the short‐circuit drift current), confirming that the penalties from the nonuniform photocarrier generation and perturbation in the flow of carriers^19^, ^20^, ^36^ are negligible in this case, where the plasmonics structures are relatively smooth.

To find the optimal (optical–electrical) structure of the CC‐UTMF OSC, different sets of *P*/4 and *A* have been tested in the range of 1000–2500 nm for the calculation of PCE. The obtained PCE was very stable (PCE difference < 0.7%) in the ranges of 1000 nm ≤ *P*/4 ≤ 2500 nm and 1000 nm ≤ *A* ≤ 2500 nm, and showed a maximum of ≈13.0% at *P*/4 = 2100 nm and *A* = 1200 nm. It is worth mentioning that the optimal structure in terms of optical absorption (*P*/4 = 2000 nm, *A* = 1900 nm) resulted in slightly lower PCE (≈12.9%, Figure S4, Supporting Information).

**Figure 4:**
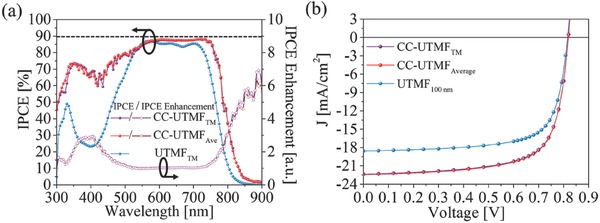
Electrical performance of the CC‐UTMF OSC. a) IPCE (solid symbols) and the IPCE enhancement ratio (open symbols) spectra relative to the IPCE of control UTMF (blue) (dashed horizontal line marking 90%). b) *J–V* characteristics of UTMF OSC and CC‐UTMF OSC. All data are taken with AM1.5G‐weighted, normally incident plane‐wave illumination.

**Figure** **4**a shows a plot of the calculated IPCE and IPCE enhancement ratios of the proposed device compared with those of the control reference. The clear improvement in IPCE over the full spectral range supports the large improvement in the short‐circuit current (≈21.0%) in the *J*–*V* curve (Figure 4b) and PCE (≈19.7%), emphasizing the significance of spectral engineering enabled by the corrugated cavity. The effect of carrier mobility in the optimal structure is also tested by using much larger value of 10 *µ*_e,h_ (Figure S9, Supporting Information). When compared to Figure 3 (*µ*_e,h_), slightly increased currents have been observed. However, since the carrier collection is already very efficient in thin active layers (<150 nm, as discussed in Figure 1d), the mobility contribution to the final PCE was much less significant (13.0–13.1%, for the mobility increase of *µ*_e,h_ to 10 *µ*_e,h_) when compared to the increased optical absorption with the corrugated cavity. The details of the overall performance of the OSCs are summarized in **Table** **1**. Further results for TE polarization can be found in the Supporting Information.

**Table 1:** Photovoltaic characteristics of UTMF control reference and corrugated cavity UTMF OSCs under AM1.5G‐weighted illumination

Case	*J*_sc_ [mA cm^−2^]	*V*_oc_ [V]	FF [%]	PCE [%]
UTMF control reference (100 nm)	18.57	0.815	71.7	10.85
Corrugated cavity UTMF (TE)	22.54	0.819	70.9	13.1
Corrugated cavity UTMF (TM)	22.38	0.819	70.3	12.9
Corrugated cavity UTMF (average)	22.46	0.819	70.6	13.0

## 3 Conclusion

In conclusion, the highest performance of an organic solar cell of ≈13.0% PCE was numerically demonstrated by identifying the limitation of conventional UTMF OSC through optical–electrical multiphysics analysis and then introducing a corrugated cavity structure which perfects the spectral engineering over the optical absorption of the ultrathin PBDTT‐F‐TT:PC_71_BM active layer. Ultralow *Q* (330–775 nm), highly uniform (>85%) coverage of optical absorption is derived from the hybridized Fabry–Perot cavity mode and multipeak in‐plane propagation modes, without any penalty in the electrical carrier transport dynamics. Compared to the flat UTMF OSC, large improvements of ≈21.5% in optical absorption, ≈21.0% in the short‐circuit current, and ≈19.7% in the PCE enhancements are confirmed from optical–electrical multidisciplinary numerical analysis, thereby realizing 13% of PCE for the first time for the OSC, notably with widely used organic materials. Clearly revealing the significance of spectral engineering, driven by the corrugated cavity, we anticipate the application of the same concept to ultrathin OSCs based on other materials, such as PBDB‐T:IT‐M for the realization of even higher PCE. Moreover, it is emphasized that the use of optical–electrical multiphysics analysis should be considered in the proper identification and overcoming strategy of the performance limiting factor (e.g., electrical transport vs optical engineering), for any type of solar cells.

## 4 Experimental Section

*Structure of the Reference UTMF OSC and Corrugated Cavity UTMF OSCs*: The reference OSC and suggested CC‐UTMF OSC assumed the same material compositions, except for the corrugation. The reference flat UTMF OSC was constructed with a structure of TeO_2_/UTMF‐Ag/FPI‐PEIE/PBDTT‐F‐TT:PC_71_BM/MoO_3_/Ag (Figure 1a). For the CC‐UTMF OSC, a smooth sine‐shape spatial modulation was introduced to derive a corrugated cavity structure (Figure 2c), where *P* is the major axis period (in‐plane) and *A* is the amplitude of the minor axis (vertical). For the efficient electrical charge transport and optical transparency, TeO_2_ was selected as a capping layer.^1^, ^6^ The thicknesses of FPI‐PEIE, MoO_3_, UTMF‐Ag, and TeO_2_ were set as 10, 6, 12, and 50 nm, respectively.

The corrugation structure in Figure 2c could be fabricated by using the self‐assembly process,^37^ PDMS‐mold process,^38^ stretching PDMS sheets method,^39^ or mechanical release PDMS method.^40^ For the case of mechanical release PDMS method, a poly(ethylene glycol diacrylate) (p(EGDA)) deposition on PDMS substrate was prepared^40^ to construct a sinusoidal corrugation. Then, the PDMS substrates were subjected to release along *x*‐axis. Putting the PDMS film in an oven at 40 °C under vacuum, sinusoidal corrugation structure could be obtained, from mechanical strain tuning. It was noted that the period and geometry of the corrugation could be controlled with the process parameters used in the fabrication of the release rate and compressive stress.

*Coupled Optical–Electrical Device Simulations*: All simulations were performed using the finite element method, implemented in COMSOL Multiphysics. The optical and electrical properties of BHJ OSCs were modeled by solving organic semiconductor equations consisting of two parts: the optical and electrical domains. First, the optical part solved Maxwell's equations for specified illumination conditions and then calculated the electric field intensity |*E*(*x*,*y*,*z*)|^2^ for the later determination of exciton generation rates. The optical absorption in the active layer was obtained as a fraction of the absorbed incident power (i.e., $A\left(\lambda \right)\;\;=\;\;P_{\mathrm{in}}^{-1}\int \frac{1}{2}\left(2\pi c/\lambda \right)\;\;\cdot \;\;\varepsilon_{2}\left(\lambda \right)\;\;\cdot \;\;{\left|E\left(x,y,z,\lambda \right)\right|}^{2}dV$) under AM1.5G illumination conditions. Optical parameters used in the calculation included complex refractive indices of PBDTT‐F‐TT:PC_71_BM, MoO_3_, FPI‐PEIE and TeO_2_, taken from ref. 1, and the dielectric permittivity of Ag, modeled by using the measured data from Palik.^41^ Second, the electrical part solved the coupled Poisson, drift‐diffusion, and charge carrier continuity equations taking the exciton generation rates as input. The electrical parameters used included band‐gap and material energy levels taken from ref. 11, 42, 43, and the electron and hole mobilities (*µ*_N_, *µ_p_*) from ref. 24, 25. The coupled Poisson, carrier drift‐diffusion, and continuity equations are given by

(1) $$\nabla^{2}\phi \;\;=\;\;-\;\;\frac{q\left(p\;\;-\;\;n\right)}{\varepsilon }$$

(2) $$\frac{\partial n}{\partial t}\;\;=\;\;D\;\;-\;\;\left(1\;\;-\;\;Q\right)R\;\;-\;\;\frac{n\;-\;n_{\mathrm{eq}}}{\tau_{n}}\;\;\;+\;\;\;\frac{1}{q}\nabla \;\;\cdot \;\;\left(qn\mu_{n}\nabla \phi \;\;+\;\;k_{B}\;T\mu_{n}\;\nabla n\right)$$

(3) $$\frac{\partial p}{\partial t}\;\;=\;\;D\;\;-\;\;\left(1\;\;-\;\;Q\right)R\;\;-\;\;\frac{p\;-\;p_{\mathrm{eq}}}{\tau_{p}}\;\;-\;\;\frac{1}{q}\nabla \cdot \left(qp\mu_{p}\;\nabla \phi \;\;-\;\;k_{B}T\mu_{p}\nabla p\right)$$

where *q* is the elementary charge, *k*_B_ is the Boltzmann constant, φ is the electrostatic potential, *D* is the Onsager–Braun dissociation (i.e., *D* = *G*_opt_ · *Q*(*x*,*y*,*z*) dissociation),^44^, ^45^, ^46^ *Q* is the local dissociation probability, *R* is the local recombination rate,^19^, ^20^, ^21^, ^44^, ^45^, ^46^, ^47^, ^48^ *µ*_N_ and *µ_p_* are the electron and hole motilities, *p* and *n* are the charge carrier densities, *p*_eq_ and *n*_eq_ are the equilibrium densities, and *τ_n_* and *τ_p_* are the lifetimes of the electrons and holes, respectively. The exciton generation rate (in the active layer) was obtained from *G*_opt_(λ) = *ε″*|*E*(*x*,*y*,*z*)|2/2*h–*, where *ε″* is the imaginary part of the dielectric permittivity, and *h–* is the reduced Planck constant. Further details of the optical and electrical simulation can be found in the previous papers,^19^, ^20^ and in ref. 21, 22, 23.

Verification of the used model in comparison to experimental results of ref. 1 (flat UTMF OSCs) can be found in Figure S10 and Table S1 in the Supporting Information, including comparisons of optical absorption/reflection, *J–V* characteristics, corresponding discussions, and list of fundamental parameters.

## Conflict of Interest

The authors declare no conflict of interest.

## Supporting information

Supplementary
