# Supplementary material for: Ultrathin Organic Solar Cells with a Power Conversion Efficiency of Over ≈13.0%, Based on the Spatial Corrugation of the Metal Electrode–Cathode Fabry–Perot Cavity
Source: Adv Sci (Weinh). 2018 Jan 31;5(4):1700900. doi: 10.1002/advs.201700900 (PMC5908355; doi:10.1002/advs.201700900)
Supplement: Supplementary file 1 — Supplementary [file ADVS-5-1700900-s001.pdf]

Copyright WILEY-VCH Verlag GmbH & Co. KGaA, 69469 Weinheim, Germany, 2018.

ADVANCED SCIENCE Open Access

Supporting Information

for *Adv. Sci.,* DOI: 10.1002/advs.201700900

Ultrathin Organic Solar Cells with a Power Conversion Efficiency of Over ~13.0%, Based on the Spatial Corrugation of the Metal Electrode–Cathode Fabry–Perot Cavity

*Sungjun In and Namkyoo Park**

Copyright WILEY-VCH Verlag GmbH & Co. KGaA, 69469 Weinheim, Germany, 2013.

Supporting Information

Title Ultrathin organic solar cells with a power conversion efficiency of over ~13.0%, based on the spatial corrugation of the metal electrode - cathode Fabry-Perot cavity

Sungjun In, and Namkyoo Park*

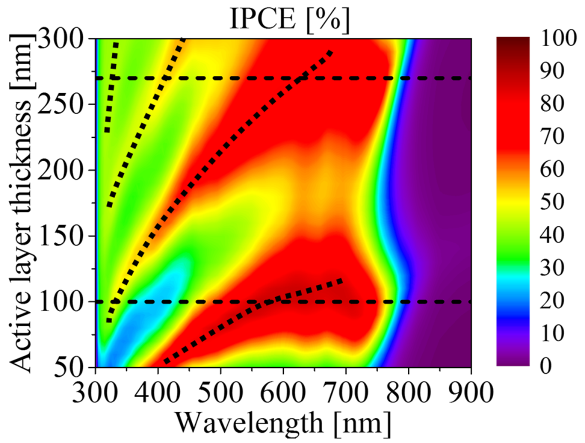

Figure S1. IPCE map of reference UTMF OSC. IPCE map as a function of active layer thickness and wavelength. In all data, AM 1.5G-weighted, normally-incident plane-wave illumination is assumed.

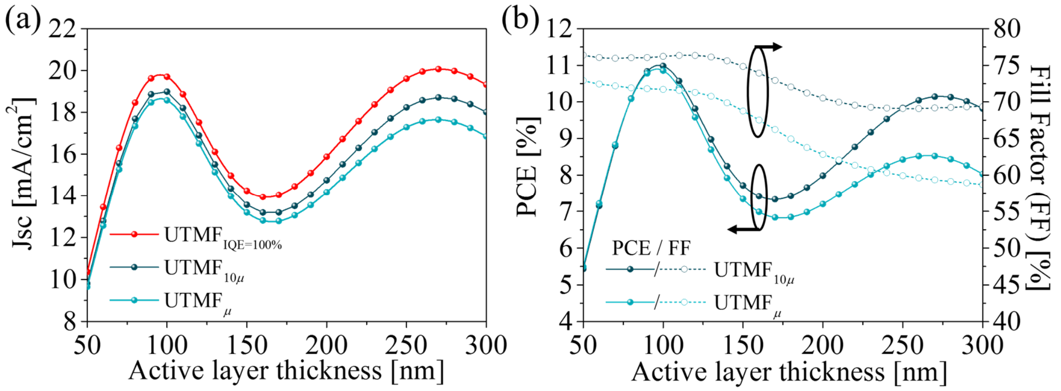

Figure S2. Thickness and mobility dependence of reference UTMF OSC. a, Ideal optical (IQE=100%) and optical-electrical coupled short circuit current densities, as a function of active layer thickness. **b,** Thickness dependence of Fill factor (FF) and PCE. In all data, AM 1.5G-weighted, normally-incident plane-wave illumination was used.

Definition of a figure of merit (FOM). Optical absorption is characterized by the figure of merit (FOM), defined as the ratio of incident photons (with AM1.5G spectral weighting) absorbed in the active layer:

$$FOM = \left(\int_{\lambda 300}^{\lambda 900} \frac{\lambda}{hc} \ I(\lambda) \ A(\lambda) \ d\lambda \right) \Big/ \left(\int_{\lambda 300}^{\lambda 900} \frac{\lambda}{hc} \ I(\lambda) \ d\lambda \right)$$

where h is the Plank constant, c is the speed of light in free space, $I(\lambda)$ is the AM1.5G solar spectrum weighting factor, $A(\lambda)$ is the optical absorption, and the integration region is the active layer inside the cavity.

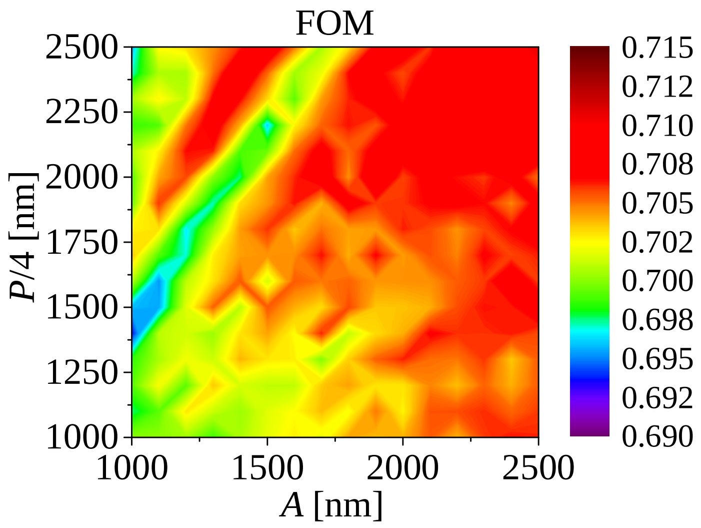

Figure S3. FOM map of CC-UTMF OSC, as a function of the corrugation period and amplitude. The optical absorption and FOM were calculated by changing the amplitude and period of the corrugation, over the ranges of 1000 nm< $P/4$ < 2500 nm and 1000 nm< A < 2500 nm.

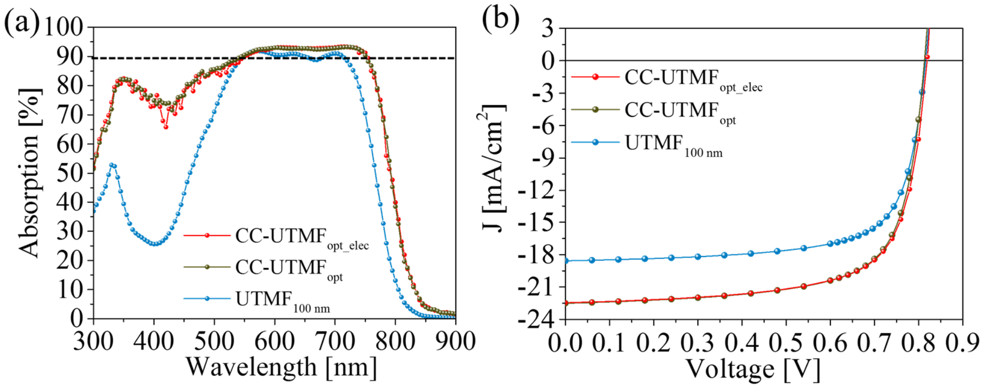

Figure S4. Optical and electrical device performance of CC-UTMF OSC at the maximum optical absorption. a, Optical absorption spectra. **b,** *J-V* characteristics. The optical optimum geometric parameters are $P/4$ = 2000 nm and A = 1900 nm (red: optical-electrical optimum, dark green: optical optimum). All data correspond to AM 1.5G-weighted, normally-incident plane-wave illumination.

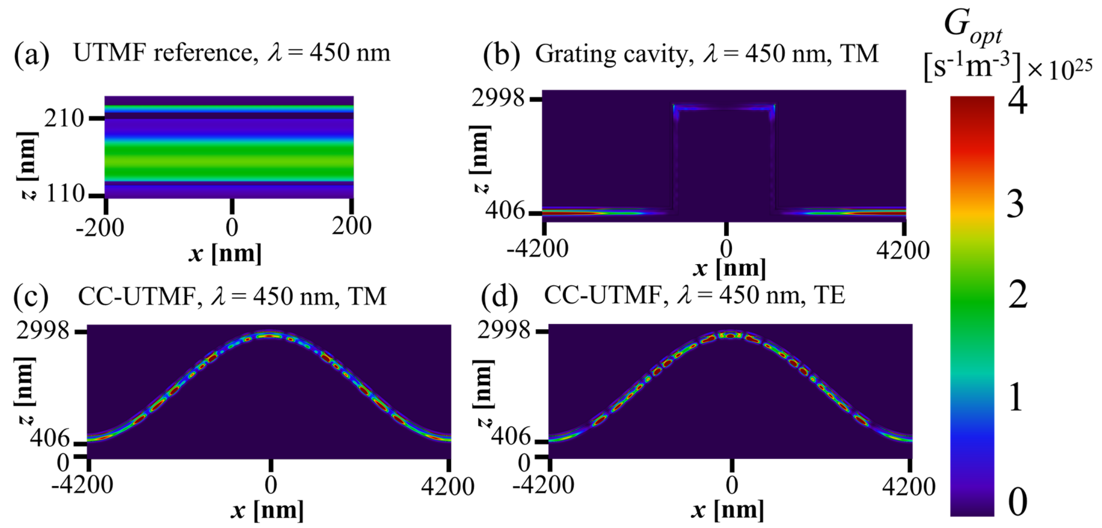

Figure S5. Distribution of exciton generation rates under AM 1.5G weighted illumination. For **a**, reference UTMF OSC, **b**, Square-grating cavity UTMF OSC, and **c, d**, CC-UTMF OSCs under TM/TE polarization. Normal-incident plane-wave illumination, λ =450 nm.

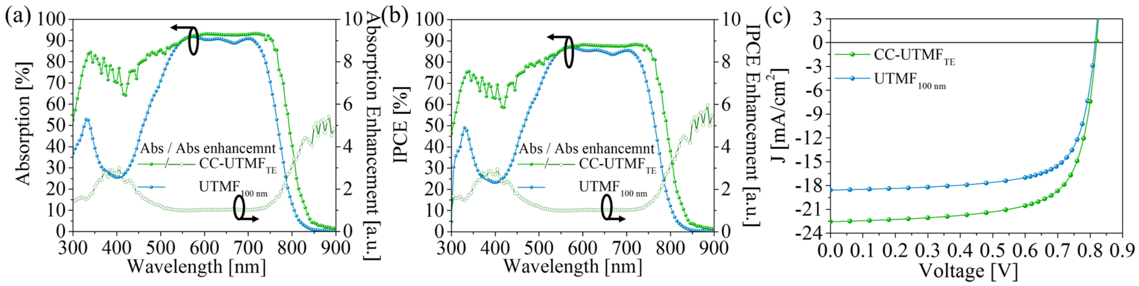

Figure S6. Device characteristics under TE polarization illumination. a, Optical absorption (solid symbols) and absorption enhancement ratio (open symbols) spectra. **b,** IPCE (solid symbols) and IPCE enhancement ratio (open symbols) spectra. **c,** *J-V* characteristics of reference UTMF OSC (blue), CC-UTMF OSCs (green). All data taken with AM 1.5G-weighted, normally-incident plane-wave illumination.

Drift current. By analyzing drift currents, it is possible to understand the effects of local mobility, internal built-in electric field, and nano structures on the electrical performance of OSCs. Current densities are given by $j_n = -n\mu_n q\nabla\phi$ and $j_p = p\mu_p q\nabla\phi$, where n and p are the carrier densities, q is the elementary charge, and ϕ is the electrostatic potential.

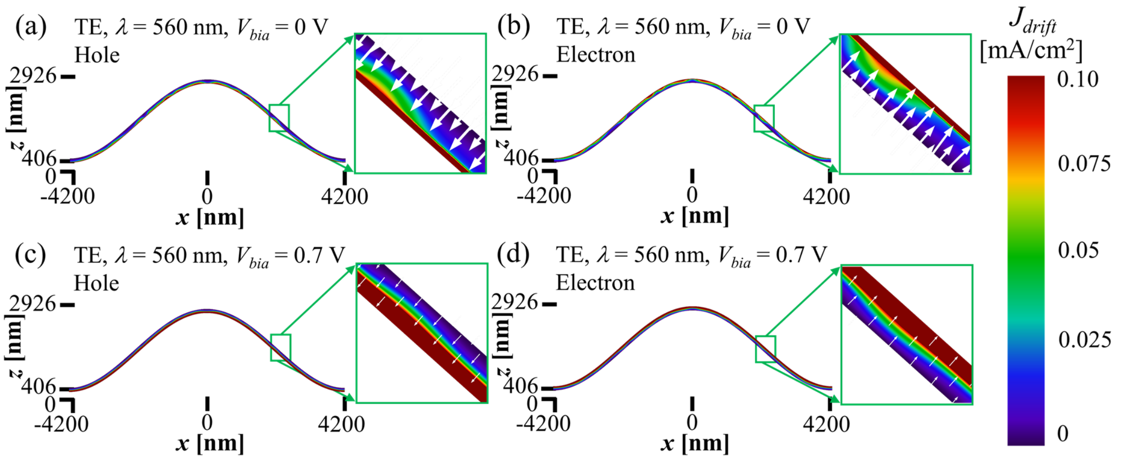

Figure S7. Drift current density and current flow of the CC-UTMF OSC, under the short-circuit current (V_{bias} = 0 V) and near open-circuit voltage condition (V_{bias} = 0.7 V). (**a, c**) Hole and (**b, d**) electron drift current density (colored bands) and current flow (white arrow) under TE polarization at λ = 560 nm.

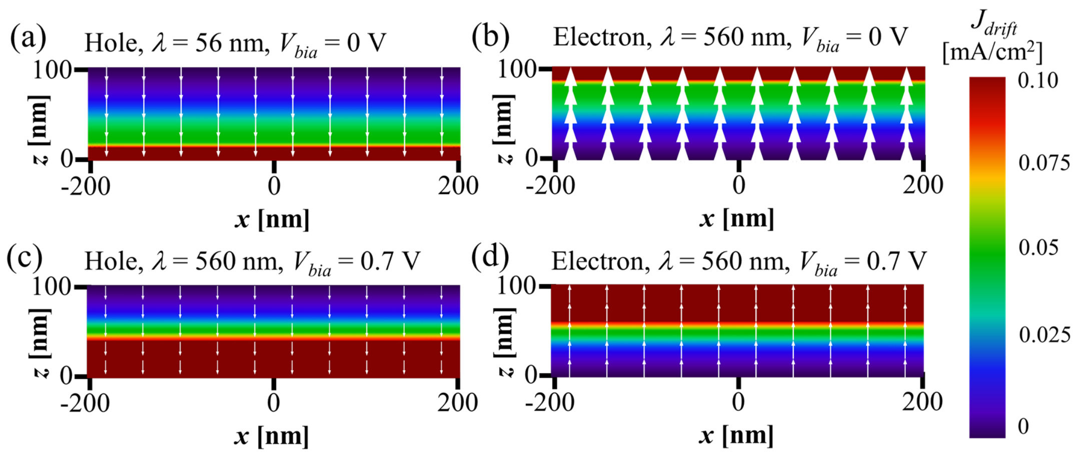

Figure S8. Drift current density and current flow of reference UTMF OSC, under the short circuit current and near open circuit voltage condition. Drift current distribution of reference UTMF-electrode OSCs at the active layer thickness of 100 nm.

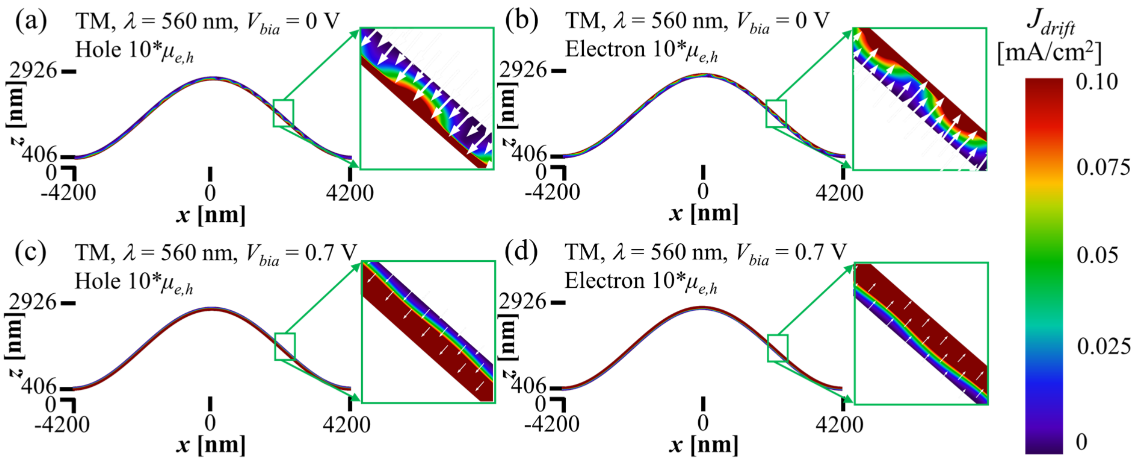

Figure S9. Drift current density and current flow of the CC-UTMF OSC with 10$\mu_{e,h}$ under short-circuit current (V_{bias} = 0 V) and near open-circuit voltage (V_{bias} = 0.7 V) condition. (**a, c**) Hole and (**b, d**) electron drift current density (color) and current flow (white arrows) of CC-UTMF, under TM polarization illumination at λ = 560 nm.

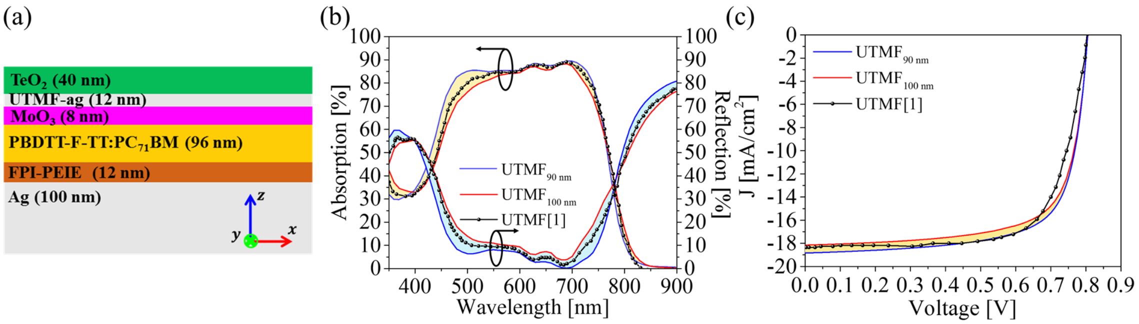

Figure S10. Stability test of the used model in comparison to experimental results in [1]. All experimental data and optical simulation parameters are taken from [1], while electrical simulation parameters are taken from [11, 21-25, 48]. **a,** Schematic of flat UTMF OSC. Comparison of numerically-obtained **b,** absorption spectra in the active layer, and numerically- and experimentally- obtained **c,** J-V characteristics of flat UTMF OSC shown in (a). For active layer thickness of 90 ~ 100 nm, the calculated PCE ranged between 10.83 % to 10.42 %, with corresponding short circuit currents between 18.83 mA/cm^2 and 18.15 mA/cm^2: all in good agreement with experimental result ([1], PCE = 10.5%, J_{sc} = 18.3 mA/cm^2 for active layer thickness = 96 nm).

Table S1. Simulation parameters used in coupled optical-electrical analysis

Parameter	Value	Reference
Electron mobility	3.42×10^{-4} [$cm^2V^{-1}s^{-1}$]	24
Hole mobility	3.58×10^{-4} [$cm^2V^{-1}s^{-1}$]	25
Relative permittivity	3.5	48
Electron diffusion constant	8.84×10^{-6} [cm^2s^{-1}]	24

Hole diffusion constant	9.25×10^{-6} [cm^2s^{-1}]	25
HOMO of PBDTT-F-TT	-5.22 [eV]	11
HOMO of $PC_{71}BM$	-5.8 [eV]	11
LUMO of PBDTT-F-TT	-3.64 [eV]	11
LUMO of $PC_{71}BM$	-4.0 [eV]	11
Doping density (N_A, N_D)	$2e^{21}$ [m^{-3}]	Fitted for experiment
Band effective densities of states (N_C, N_V)	$9.81e^{23}$ [m^{-3}]	Fitted for experiment
